# Loss of socioemotional and occupational roles in individuals with Long COVID according to sociodemographic and clinical factors: Secondary data from a randomized clinical trial

**DOI:** 10.1371/journal.pone.0296041

**Published:** 2024-02-22

**Authors:** Sandra León-Herrera, Mario Samper-Pardo, Bárbara Oliván-Blázquez, Raquel Sánchez-Recio, Rosa Magallón-Botaya, Rafael Sánchez-Arizcuren

**Affiliations:** 1 Department of Psychology and Sociology, University of Zaragoza, Zaragoza, Spain; 2 Institute for Health Research Aragón (IIS Aragón), Zaragoza, Spain; 3 Department of Health Sciences, University of Zaragoza, Zaragoza, Spain; 4 Department of Preventive Medicine and Public Health, University of Zaragoza, Zaragoza, Spain; 5 Department of Medicine, University of Zaragoza, Zaragoza, Spain; 6 Department of Physiatry and Nursing, University of Zaragoza, Zaragoza, Spain; Federal University of Rio Grande do Norte: Universidade Federal do Rio Grande do Norte, BRAZIL

## Abstract

**Background:**

Long COVID syndrome can have a major impact on life organization. Its persistent symptoms may cause a potentially disabling condition that affects the quality of life of those suffering from it. The resulting loss of functional independence hinders the ability to return to normal life. Many research studies carried out on this novel syndrome have focused on describing its extensive symptomatology. Studies on later repercussions, however, such as disability or loss of significant roles, remain scarce. This study examines the loss of socioemotional and occupational roles experienced by individuals suffering from Long COVID, as a result of the disease. A secondary objective is to analyze the sociodemographic and clinical factors associated with this loss of roles.

**Patients and methods:**

A cross-sectional study was conducted with the participation of 100 patients diagnosed with Long COVID, over the age of 18, and attended by Primary Health Care in the Autonomous Community of Aragon. The main study variable was the loss of significant socioemotional and occupational roles by the participants. Sociodemographic and clinical data were also collected through a structured interview. Subsequently, a descriptive, correlational, and regression-based statistical analysis was performed using the SPSS Statistics program.

**Results:**

Based on the 100 study participants, the median number of roles lost was 3 (IQR 2) and the median number of valuable roles lost was 2 (IQR 2). More cognitive impairment and not having an active work role were predictors of a greater loss of valuables roles.

**Conclusion:**

Long COVID symptoms hinder the development of socioemotional and occupational roles. Healthcare professionals should consider this when intervening to ensure that their patients may recover their life as it was before the disease.

## Introduction

The disease caused by the severe acute respiratory syndrome, SARS-CoV-2 (known as COVID-19) has had a considerable impact globally, resulting in a devastating health, social and economic crisis [1].

The virus’ consequences on health have been highly varied, from patients who are asymptomatic to those in a life-threatening situation [2]. Although most infected people recover from the varied symptoms of the disease, some of these symptoms, such as dyspnea and fatigue may persist for several months after recovery. This has been found in approximately 50% of the cases. Other persistent symptoms such as stress, anxiety, and neurological or cognitive impairment have also been associated with long-term disease [3]. In addition to the above, generalized body aches, chest pain, elevated body temperature, palpitations, or muscle aches, among others, may also persist [4]. This condition, in which patients continue to experience symptoms even months after the acute phase of the disease has passed, was defined by the World Health Organization in October 2021 as “Post COVID Condition” [5]. In the scientific field, however, it is also often referred to as “Long COVID” or “Post-acute sequelae of COVID 19 (PASC)” [6].

Ongoing research has suggested that Long COVID should be recognized as a potentially disabling condition since its symptoms may affect the quality of life of those suffering from it [7]. The loss of functional independence resulting not only from the persistent physical symptoms after infection but also from the cognitive impairment and mental health problems such as anxiety or depression, which affect emotional well-being, hinder the return to normal life [8,9].

Long COVID syndrome may have an impact on people´s life organization and social roles. They may develop limitations in activities of daily living (ADL) such as walking, bathing, or dressing, resulting in a deteriorated functional status [10]. In addition to the impact on basic activities, limitations may also arise in occupational, social, or leisure activities [11]. According to a survey conducted by the Spanish Society of General and Family Physicians (SEMG), Long COVID syndrome may have repercussions at a work, familial, and social level, three of the main roles played by individuals [12].

Roles are defined as how individuals organize their time to satisfy their personal needs or other types of social pressure. Some of these roles are: family, friend or spouse, student, or worker. All of these roles begin to be established during adolescence or early adulthood [13]. Playing these roles allows for the possibility of connecting people, providing behavioral guidance, influencing daily health behaviors, and offering a sense of control over one’s life. This generally leads to positive outcomes in terms of physical and mental health, as well as quality of life [14,15]. Conversely, a person’s difficulties, limitations and restrictions in participating in their social roles is referred to as a disability and is one of the most relevant determinants of health and quality of life [16].

Individuals suffering from Long COVID tend to experience a major disability in situations such as work performance, maintaining relationships, caring for family members, or engaging in physical exercise. This may lead to social isolation, stigmatization, or the loss of social identity [7]. This in turn leads to the need to return to their previous quality of life and regain the roles that have been lost [17]. Gender, an important sociodemographic factor, may influence the modification or loss of activities and roles in this population since Long COVID is more likely to affect women than men [2]. In addition, there are differences in gender roles as defined by society and these tend to be exacerbated in times of crisis, such as fighting a disease [18,19].

Most research studies on Long COVID have focused primarily on the description of its extensive symptomatology. Studies on subsequent repercussions, such as disability or the loss of significant roles, however, are scarcer. Conceptualizing the disability caused by this disease and its associated factors is important to establish assessment and treatment tools based on distinct rehabilitation approaches [20,21].

The objective of this study was to analyze the loss of socioemotional and occupational roles experienced by individuals suffering from Long COVID as a result of the disease. As a secondary objective, the sociodemographic and clinical factors related to this loss of roles were analyzed.

## Materials and methods

### Study design

This was a cross-sectional study analyzing data from the baseline assessment of a randomized clinical trial entitled *“Analysis of the symptoms and quality of life of people with a diagnosis of long COVID-19*, *and the effectiveness of an intervention in primary care using ICTs”* (reference number ISRCTN91104012) [22–24].

### Study participants

The study population consisted of individuals experiencing Long COVID symptoms who were 18 years of age or older and were under treatment by primary health care professionals. Exclusion criteria were: the diagnosis of a serious uncontrolled illness, which could interfere with the clinical trial intervention; pregnancy and breastfeeding; significant risk of suicide; participation in another clinical trial within the last six months; existing structured rehabilitative or psychotherapeutic treatment conducted by healthcare professionals, or the presence of any medical, psychological, or social problems that could significantly interfere with the patient’s participation in the study.

### Sample size and sampling procedure

Given that this was a secondary study, the sample size had been calculated to respond to the main objective of a randomized clinical trial (RCT): to analyze the effectiveness of an intervention using a mobile APP on the quality of life of patients with Long COVID [22,24]. The total sample size required in the RCT was 78 subjects. However, due to the demand of potentially interested participants, the researchers agreed to accept approximately 28% more participants, and the final sample size consisted of 100 participants.

To ensure that this size was adequate to respond to the study objective, the required sample size was calculated based on data from Placeres et al. (2021) [25], which considers the issue of loss of roles after a disabling disease. Based on this study, with a confidence level of 95%, an accuracy of 8%, and a role loss rate of 83%, the necessary sample size was 85 subjects. An additional 10% was added to cover the possibility of incomplete questionnaires, making 94 subjects being the study’s required sample size. The sample consists of 100 participants, and thus it exceeds the required sample size.

Of these participants, 20 were men and 80 were women. Primary health care (PHC) professionals participating in the project within a PHC environment were responsible for recruiting patients and future study participants. Patients who belonged to the Long COVID Association of Aragon also participated. Recruitment was performed consecutively until the sample size was achieved. It extended over 3 months, from January to March 2022.

### Data collection procedure

Sociodemographic, clinical, and other data were obtained directly from the information provided by patients during the clinical trial’s baseline interview, using an ad-hoc questionnaire. All data collected were processed according to current regulations on data protection (Organic Law 3/2018, of 5 December, on the protection of personal data and guarantee of digital rights).

### Variables and measures

The main variables of the study were the number of lost roles and the number of valuable roles lost. Roles were also collected using the Spanish version of the Role Checklist, with a test-retest reliability (measured by weighted Kappa) of 0.74 [26]. This is a two-part inventory. The first part measures the presence of the ten most important roles in people’s lives over time. It also adds a section called “Other roles” to include any other relevant role for the evaluated individual. Participants are asked to indicate whether they have performed each of the roles in the past (at any time up to the week immediately preceding the assessment), whether they are currently performing them (on the day of checklist completion and for the previous seven days), and whether they plan or wish to perform them in the future (at any time as of the day after the assessment). It is possible to tick each role more than once. The second part measures the value that the individual attributes to each role (“Not at all valuable”, “Somewhat valuable”, or “Very valuable”). Participants mark the value that they consider for each of the roles, even if they have never played them or do not plan to do so in the future [27].

Regarding the secondary study variables, the following were collected:

- Socio-demographic variables: sex, age, marital status, level of education, and occupation.
- Clinical variables: number of residual symptoms and their severity measured via an Analogue Visual Scale [28]. Residual symptoms include: gastrointestinal symptoms, loss of smell or anosmia, loss of taste or ageusia, blurred vision, eye problems, tiredness or fatigue, cough, low-grade fever (37°C—38°C), fever (above 38°C), chills or shivering without fever, bruising, myalgia, headaches, sore throat, dyspnoea, chronic fatigue, dizziness, tachycardia, orthostatic hypotension, joint pain, chest pain, back pain, neurological symptoms, memory loss, confusion or brain fog, short attention and concentration spans, loss of libido or erectile dysfunction, altered menstrual cycle, urinary symptoms, hair loss, and other “residual” symptoms [29–31].
- Cognitive variables: To assess the presence of cognitive impairment, the official Spanish version of the Montreal Cognitive Assessment (MoCA) [32,33] was used. This test has an adequate internal consistency (Cronbach’s alpha of 0.76) and assesses six cognitive domains (memory; visuospatial ability; executive function; attention, concentration or working memory; language, and temporospatial orientation). It is based on a total of 30 points, and it is possible to make a correction of one point in the case of subjects with less than 12 years of schooling. In its original version, the cut-off point for the detection of mild cognitive impairment is 26 [34]. This test has already been used to assess the cognitive impairment of people with long COVID, [35,36]. The Cronbach’s alpha obtained in this study was 0.373.
- Physical functioning variable: It was measured using the Sit-to-Stand Test [37]. 30-second Sit-to-Stand Tests were used. They are specifically used to detect respiratory pathologies [38]. The test assesses resistance at a high power, speed, or velocity in terms of muscular or strength resistance by recording the number of times an individual can fully stand up and sit down during a 30-second period. It has good test-retest reliability (.84 <R< .92), has been translated into Spanish, and has been previously used in patients with COVID-19 [39].
- Affective state: It was assessed using the Hospital Anxiety and Depression Scale (HADS) questionnaire [40]. This self-reporting scale was developed to screen for depression and anxiety disorders in medical patients in primary care settings. It consists of 14 items divided into two subscales that separately assess anxiety and depression (HADS-A and HADS-D, respectively). Each item is rated on a 4-point scale (zero to three), with total scores ranging from 0 to 21 for symptoms of anxiety and depression (0 to 42 for total score). Higher scores indicate more severe symptoms. The HADS has been translated into several languages, including Spanish [41], to facilitate its use in international trials [42]. The Cronbach’s alpha obtained in this study is 0.91.
- Sleep quality: The Insomnia Severity Index (ISI) was used to measure the sleep quality of the study participants. This scale [43] consists of self-reports that measure the patient’s perception regarding nocturnal and diurnal symptoms of insomnia: difficulties falling asleep, staying asleep, waking up early in the morning, satisfaction with current sleep pattern, consequences of sleep quality in daily functioning, noticeability of impairment attributed to sleep deprivation by the immediate social environment, and degree of distress or concern caused by the lack of sleep. Each of these seven items has a response option ranging from zero to four, and the general score obtained ranges from 0 to 28. High scores indicate greater severity of insomnia. The Spanish version of the index [44] has adequate internal consistency (Cronbach alpha = 0.82). The Cronbach’s alpha was 0,86 in this study. This scale has been previously used in other studies on individuals with Long COVID [45].
- Social support: The official Spanish version [46] of the Medical Outcomes Study Social Support Survey (MOS-SS) was used to measure the social support of study participants. This self-reporting instrument consists of four subscales (emotional/informational, tangible, affectionate, and positive social interaction) with an overall functional social support index. It is quite stable over time and has good reliability (Cronbach’s alpha ≥0.91). The Cronbach alpha obtained in this study was 0.94. The scale consists of 19 items, with a 5-point Likert Scale. Higher scores indicate more social support [47].

### Statistical analysis

The IBM SPSS Statistics V22.0. and Microsoft Excel software were used to perform the statistical analyses for this study. First, the sample distribution was analyzed using the Kolmogorov-Smirnov statistic. Values were found to be under 0.05 for all variables except for the number of symptoms. Therefore, non-parametric statistics were used. Subsequently, a descriptive analysis was performed, using the median and interquartile range for continuous variables, and frequencies and percentages for categorical variables. Gender-based differences in the above variables were compared using the Mann–Whitney U and chi-square tests. A bivariate analysis was performed, analyzing the number of valuable roles lost according to gender, marital status, educational level, and occupation, using the Mann-Whitney U test. Alternately, a correlation analysis was performed between the number of valuable roles lost and the continuous variables collected (age, number of persistent symptoms, months since infection, Montreal Cognitive Assessment, Sit-to-Stand Test, affective state, Insomnia Severity index, and social support) using Spearman’s Rho statistic. A multivariable model was employed including the number of lost valuable roles as the dependent variable and the other significative variables as independent variables (age, active-non active work role, number of persistent symptoms, Montreal Cognitive Assessment, Sit-to-Stand test). To obtain a final model, the independent variables were added to the regression model [48]. A linear regression was performed since the residuals of the model had a normal distribution, finite mean, and constant variance. However, a bootstrapping analysis was also conducted with 2000 samples. All levels of significance were established at 0.05.

### Ethical consideration

The Ethics Committee for Clinical Research of Aragon Ethical granted ethical approval for this study (PI21/454). All procedures to be carried out in this work complied with the ethical standards of this committee and with the Declaration of Helsinki of 1975. All participants signed a written informed consent form, and their data were anonymized and used solely for research purposes.

## Results

Of the 100 participating individuals, 80 were women and 20 were men. The median age of the participants was 47 years (IQR 11 years, range: 29–72). Table 1 presents the total sample description and a gender-based comparison, using the collected variables. The participant profile was female, married, approximately 47 years of age, with a high school or university education, and having lost 3 roles, two of which were valuable. The median number of persistent symptoms was 16.5 (IQR 8). Median scores on the cognitive assessment and physical functioning indicated an affectation in physical and cognitive functioning. According to gender, significant (or very close to significant) differences were found in education level, occupation, number of persistent symptoms, and cognitive assessment. A higher percentage of women had secondary school or university studies, more men were on sick leave, and men had lower cognitive assessment scores and a lower number of persistent symptoms. The median number of roles lost was 3 (IQR 2) and the median number of valuable roles lost was 2 (IQR 2).

**Table 1:** Description of sociodemographic and clinical variables of the total sample and gender-based comparison.

Variables	Total sample N (%) median (IQR) / mean (SD)	Men N (%) median (IQR)/ mean (SD)	Women N (%) median (IQR)/ mean (SD)	p-value
**Gender** Men Women	20 (20%) 80 (80%)			
**Age**	47 (11) / 48.2 (9.2)	49.5 (8.75) / 48 (8.3)	47 (14) / 48.35 (9.5)	0.918
**Marital status** Married or in a relationship Single, separated, widowed	70 (70%) 30 (30%)	15 (75%) 5 (25%)	55 (68.8%) 25 (31.2%)	0.585
**Educational level** Primary studies Secondary or university studies	9 (9%) 91 (91%)	4 (20%) 16 (80%)	5 (6.3%) 75 (93.7%)	0.055
**Occupation** Employee Unemployed TWD Retired Others	46 (46%) 5 (5%) 37 (37%) 9 (9%) 3 (3%)	5 (25%) 0 13 (65%) 2 (10%) 0	41 (52.6%) 5 (6.4%) 24 (30.8%) 7 (9%) 1 (1.2%)	0.059
**Number of roles lost**	3 (2) / 3.1 (1.7)	3 (2) / 3 (2)	3 (2) / 3.1 (1.7)	0.979
**Number of valuable roles lost**	2 (2) / 1.75 (1.3)	1 (1.75) / 1.65 (1.3)	2 (2) / 1.7 (1.3)	0.632
**Number of persistent symptoms**	16.5 (8) / 16.4 (5.9)	14 (13.25) / 13.8 (6.5)	17 (8.75) / 17.1 (5.7)	0.058
**Months since the infection**	17 (9.75) / 16.1 (6.3)	15.5 (9.25) / 14.6 (6.4)	17 (8) / 16.5 (6.2)	0.272
**Montreal Cognitive Assessment (MoCA)**	24 (4) / 23.6 (3.85)	22 (6.25) / 22.1 (4.6)	25 (3) / 24 (3.5)	0.068
**Sit-to-Stand Test**	10.5 (4) / 10.3 (3.4)	10 (4) / 10 (3.5)	11 (4) / 10.4 (3.4)	0.621
**Affective state (HADS)**	16 (12)	20 (16) / 18.45 (9.9)	16 (11.5) / 17.4 (7.9)	0.685
**Insomnia Severity Index (ISI)**	10.5 (11) / 11.3 (6.5)	12 (10.5) / 13.1 (7.1)	10 (11.5) / 10.9 (6.4)	0.229
**Social support (MOS-SS)**	91 (19) / 83.8 (16.3)	92,5 (18.25) / 83.65 (18.4)	91 (29) / 83.8 (15.8)	0.692

Notes: The Chi-square test was used for qualitative variables (gender; marital status; educational level; occupation). The other (quantitative) variables were compared using the Mann-Whitney U test.

TWD: Temporary work disability, MoCA: Montreal Cognitive Assessment, HADS: Hospital Anxiety and Depression Scale, ISI: Insomnia Severity Index, MOS-SS: Medical Outcomes Study Social Support Survey

Tables 2 and 3 show the relationship between the number of valuable roles lost and the sociodemographic and clinical variables. It is evident that a relationship exists between the number of valuable roles lost and age, occupation, number of persistent symptoms, Montreal Cognitive Assessment score, and Sit-to-Stand Test score. Having a higher age and number of persistent symptoms were associated with more valuable roles lost. On the other hand, lower scores on cognitive assessment and physical functioning were associated with a higher loss of valuable roles.

**Table 2:** Analysis of the number of valuable roles lost according to gender, marital status, educational level, and occupation.

Variables	Number of valuable roles lost Median (IQR) / Mean (SD)	p-value
**Gender** Men Women	1 (1.75) / 1.65 (1.3) 2 (2) / 1.7 (1.3)	0.632
**Marital status** Married or in a relationship Single, separated, widowed	2 (2) / 1.7 (1.35) 2 (2) / 1.6 (1.2)	0.670
**Educational level** primary studies Secondary or university studies	2 (1) / 1.8 (1.2) 2 (2) / 1.7 (1.3)	0.771
**Occupation** No active work role Active work role	2 (2) / 2.1 (1.4) 1 (2) / 1.1 (0.9)	**<0.001**

Note: Mann-Whitney U was used.

**Table 3:** Relationship between the number of valuable roles lost and age, number of persistent symptoms, Montreal Cognitive Assessment, Sit-to-Stand Test, affective state, Insomnia Severity Index, and social support.

Variables	Spearman Rho coefficient	p-value
**Age**	0.225	**0.024**
**Number of persistent symptoms**	0.289	**0.004**
**Months since the contagion**	-0.096	0.342
**Montreal Cognitive Assessment**	-0.303	**0.002**
**Sit-to-Stand Test**	-0.301	**0.002**
**Affective state (HADS)**	0.148	0.142
**Insomnia Severity Index**	0.167	0.097
**Social support (MOS-SS)**	-0.032	0.749

Note: Spearman’s rho coefficient was used.

Linear regression model results are shown in Table 4. Gender was not a significant variable associated with higher loss of valuable roles. The predictors of a higher loss of valuable roles were having greater cognitive impairment and not having an active work role. The models explain the 22.2% variance [R^2^ = 0.222; R^2^ adjusted = 0.172; F(5,77) = 4.405, p = 0.001]. The value of the Variance Inflation Factor (VIF) is close to 1 and far from 5 in all the variables analyzed, that is, there is practically no collinearity between them.

**Table 4:** Linear regression models regarding the number of valuable roles lost.

Number of valuable roles lost	Coefficient	p-value	Confidence interval 95%		Collinearity statistics	
			Inferior	Superior	Tolerance	VIF
constant	1.688					
Age	0.028	0.131	-0.009	0.064	0.938	1,066
Number of persistent symptoms	0.035	0.147	-0.013	0.084	0.906	1.104
Having an active work role	-0.726	**0.014**	-1.298	-0.154	0.788	1.269
Montreal Cognitive Assessment	-0.073	**0.054**	-0.146	0.001	0.894	1.118
Sit-to-Stand Test	0.017	0.672	-0.064	0.098	0.773	1.293
R2	0.222					
R2adj	0.172					

## Discussion

To the best of our knowledge, this is the first study examining the loss of socioemotional and occupational roles in individuals suffering from the Long COVID syndrome. This novel illness is highly heterogeneous and results in distinct clinical symptoms, depending on the specific patient [49]. Therefore, its impact on the life of each individual in terms of their organization and roles also differs. It is important to determine how this pathogen may affect the performance of social roles in order to offer more personalized interdisciplinary treatments and help patients recover their previous quality of life [50].

The results of this study reveal the existence of a loss of valuable socioemotional and occupational roles in patients experiencing the symptoms of Long COVID. In their study, Da Da Silveira et al. (2022) stated that the persistence of symptoms such as fatigue and muscle weakness for up to 6 months could affect the performance of certain activities of daily living [51]. Awayemi et al. reflected on the impact of long COVID on the health of those suffering from this often paralyzing and life-changing syndrome. Among other consequences, they describe a conflict in the performance of social roles [52]. Nielsen et al. (2022) also referred to the negative impact of Long COVID on activities of daily living, the ability to work, or the performance of some social roles such as being a parent, caregiver, or employee [53].

Although Long COVID itself may not be a disability, its effects are potentially limiting, and the inability to carry out the activities mentioned above can lead to a loss of social identity, stigmatization, and social isolation. These factors may negatively influence the recovery process [7] and may ultimately reduce the patient’s quality of life. Rehabilitation programs may be essential in reducing this long-term disability [54].

When analyzing the factors associated with this loss of roles, it was found that not being actively employed was a significant predictor. Work is essential in people’s lives and losing it has consequences that extend beyond financial insecurity. Employment provides daily structure and a sense of value and social engagement, while also being associated with improved mental well-being [55]. Losing one’s job, even for a short period of time, or a reduction in productivity, may have negative effects on the individual’s health and well-being. This highlights the need for strategies and programs to promote the return to employment of people with Long COVID, similar to those already developed for other chronic diseases [56].

Differentiating by gender, although it was found that women presented a higher number of persistent symptoms, there was a higher percentage of men on sick leave. Other studies examining Long COVID sick leave have also shown significantly higher proportions of men [57]. Other studies, however, have found that women take longer to return to work after a COVID-19 infection [58] and a discrepancy appears to exist regarding this issue. Therefore, it would be interesting to conduct studies that confirm or reject the hypothesis developed from this research that women take less sick leave for Long COVID than men and the possible explanatory causes for this.

The work role was not the only role that was modified. Other roles of great value to both women and men have also been altered. The Long COVID Patient Care Clinical Guide already reported the existence of a disability associated with the severity of these symptoms, visible in activities related to personal hygiene, home care, family obligations, or the enjoyment of recreational activities [17].

These changes may be correlated to factors such as older age and a higher number of persistent symptoms, especially those that would cause impairment in physical functioning and cognitive status. Research examining the impact of Long COVID has found that those with 30 or more persistent symptoms had higher levels of disability and a lower quality of life [59]. Other studies also associated cognitive and physical symptoms (cognitive deterioration, fatigue, dyspnea, or muscle aches) with an impact on the daily functioning of Long COVID patients [60,61].

### Limitations and future research directions

Currently, no prior studies have examined the modification of socioemotional or occupational roles in individuals suffering from Long COVID. Therefore, this is a novel study that sheds light on a pathology affecting all spheres of the individual. However, this study has certain limitations. When assessing the modification or loss of socioemotional and occupational roles, it was perceived that the participants had some difficulty in identifying their own roles. In other words, they were aware of the different activities that they engaged in on a daily basis prior to and during the illness, but they were unable to frame them within a specific role, given that they had not received prior training on these terms.

This study affirmed role modification in individuals suffering from Long COVID and even provided insight into these changes by differentiating according to gender. However, it may be interesting to further examine the influence of other variables, such as different age groups, ethnicities, societies, or population groups. As mentioned above, the question of occupational roles and gender differences should be further explored with regard to aspects such as taking sick leave or returning to work, since a discrepancy exists in the studies that have been published on this topic.

### Practical implications

These findings could help health and social care professionals develop improved management plans to support the recovery of Long-term COVID patients. Understanding how people’s life organization and roles have been modified by the wide range of persistent symptoms is important when developing action plans from a holistic perspective. This involves consideration of which spheres of their life have been affected and attempting to recover their pre-illness quality of life to the greatest extent possible.

## Conclusions

The symptomatology of Long COVID hinders the development of socioemotional and occupational roles. This leads to a reduction in the quality of life of these individuals and the presence of feelings such as a loss of social identity, stigma, or social isolation, factors that hinder the recovery process. Social and healthcare professionals should consider this when intervening to enable their patients to recover their life before the disease.

## Abbreviations

PASC: Post-acute sequelae of COVID 19
ADL: activities of daily living
SEMG: Spanish Society of General and Family Physicians
ICTs: Information and communication technologies
PHC: Primary health care
MoCA: Montreal Cognitive Assessment
HADS: Hospital Anxiety and Depression Scale
HADS-A: Hospital Anxiety and Depression Scale–anxiety subscale
HADS-D: Hospital Anxiety and Depression Scale–depression subscale (HADS-D)
ISI: Insomnia Severity Index
MOS-SS: Medical Outcomes Study Social Support Survey
IQR: Interquartile range
TWD: temporary work disability
